# Generalized Eruptive Histiocytosis: A Case Report in an Elderly Person With a Positive Rheumatoid Factor

**DOI:** 10.7759/cureus.94927

**Published:** 2025-10-19

**Authors:** Enas Attia, Kirkham Nigel

**Affiliations:** 1 Dermatology, Faculty of Medicine, Ain Shams University, Cairo, EGY; 2 Dermatology, Ain Al Khaleej Hospital, Abu Dhabi, ARE; 3 Pathology, National Reference Laboratory, Dubai, ARE

**Keywords:** dermatopathology, generalized eruptive histiocytosis, non-langerhans histiocytosis, non-x histiocytosis, pathology, rosai-dorfman disease

## Abstract

We report a case of generalized eruptive histiocytosis (GEH) in a 63-year-old woman with unique clinical and histopathological characteristics. She presented with a papular eruption, mainly on the dorsa of the hands and forearms. Histopathology showed a nodular dermal histiocytic lesion positive for S100, CD45, and CD163 staining in larger histiocytes. EMA, CAM 5.2, and AE1/AE3 stains were negative. We discuss the differential diagnoses and stress that GEH is usually benign, with, in this case, spontaneous regression. However, cases should be evaluated carefully for the possibility of underlying disease, and follow-up is mandatory to evaluate their evolution into other types of more aggressive histiocytosis.

## Introduction

Generalized eruptive histiocytosis (GEH) is a very uncommon, generalized, usually benign, self-limiting non-X histiocytosis [1]. It is characterized by recurrent eruptions of asymptomatic, small, firm, tan to reddish papules, symmetrically distributed over the face, trunk, and proximal limbs [1-3]. It may regress spontaneously, with residual hyperpigmentation [3]. Mucous membrane involvement is extremely uncommon, and visceral involvement has not been reported previously [1,2]. Histopathological examination demonstrates a monomorphous collection of benign histiocytes, lacking lipids, iron, or mucin deposition [2,3].

## Case presentation

**Figure 1:**
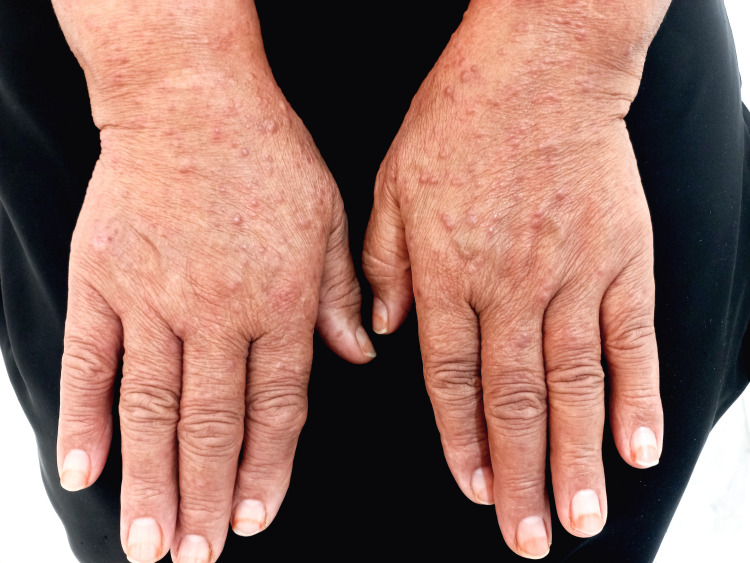
Clinical photo (before) Clinical picture at baseline: discrete flesh-coloured papules on the dorsa of both hands.

A 63-year-old female hypertensive diabetic presented to the dermatology clinic in March 2023, with extensive disseminated papulonodular lesions all over the body, which were more pronounced on both hands and forearms (Figure 1), for more than one month duration. In January 2023, she had a fever, sore throat, and cough, with joint pain, and an associated transient proteinuria. She did not experience any itching.

**Figure 2:**
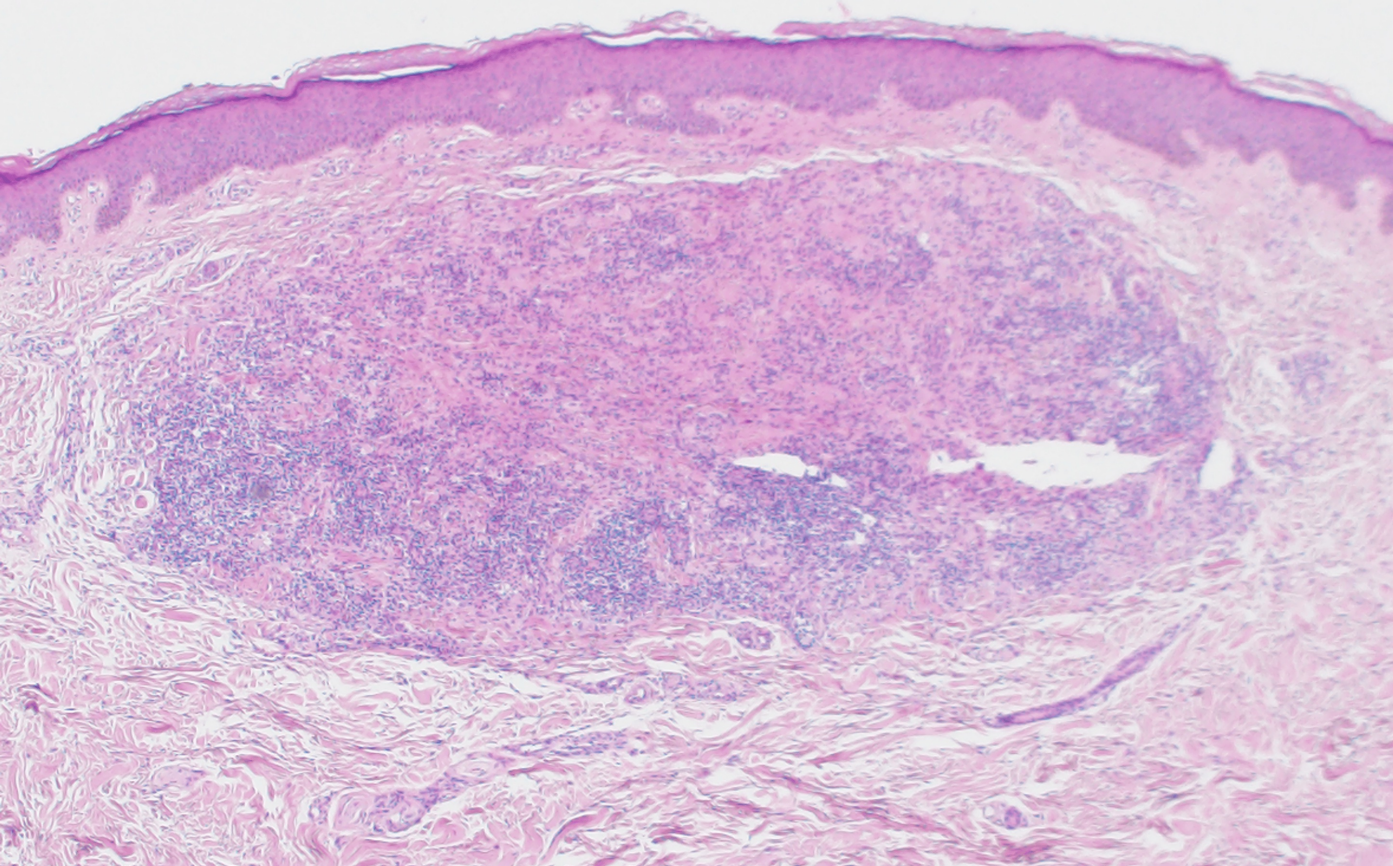
Histopathological picture (low magnification) A circumscribed nodular dermal lesion 2 x 1 mm at low power (H&E x40).

**Figure 3:**
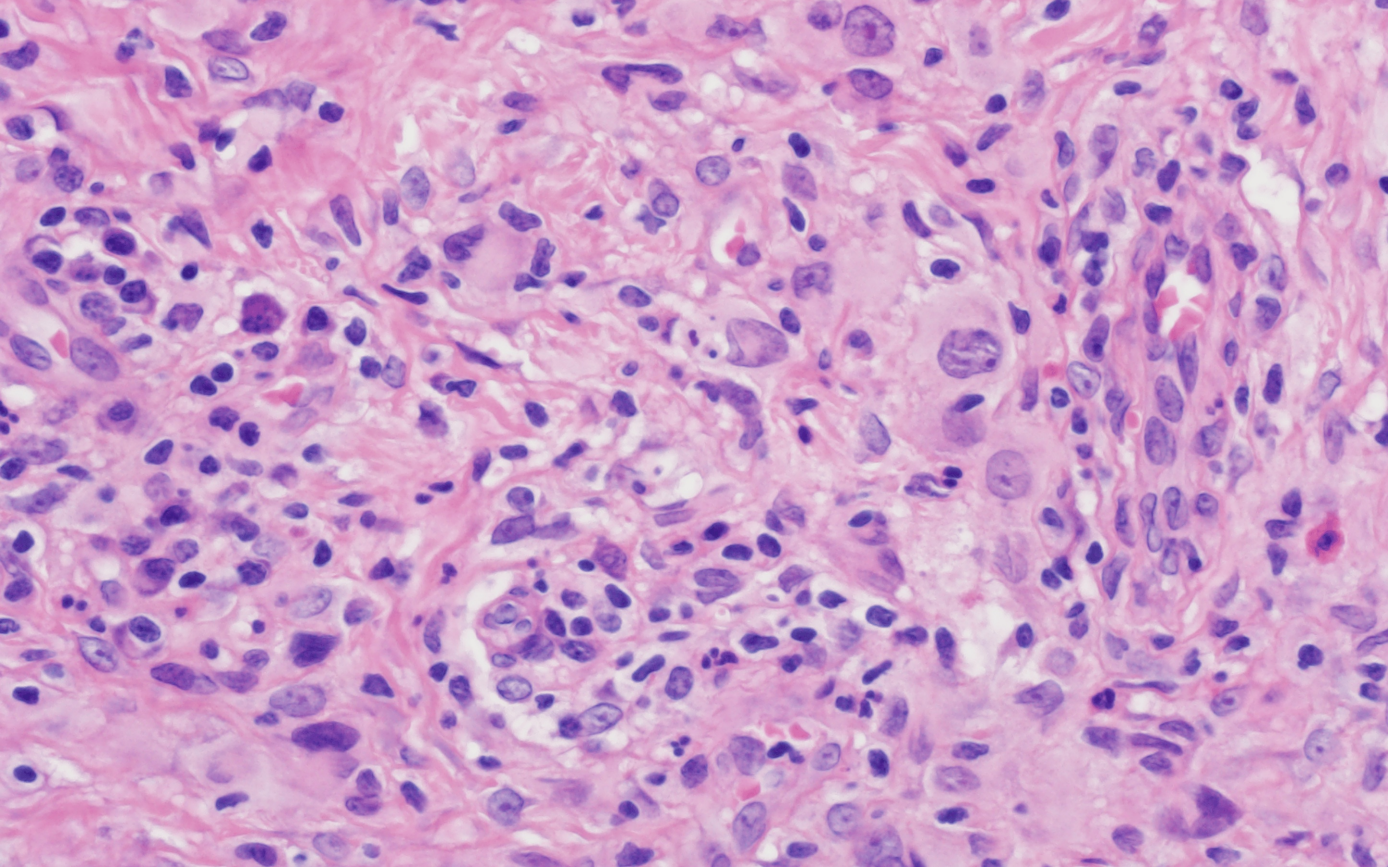
Histopathology picture (high magnification) At higher magnification, the granuloma is composed of histiocytes with medium-sized epithelioid nuclei and pale eosinophilic cytoplasm, together with lymphocytes and occasional eosinophils (H&E x 400).

A punch skin biopsy from a left forearm lesion showed a well-circumscribed nodular lesion in the upper reticular dermis with normal overlying papillary dermis and epidermis (Figure 2). The lesion was composed of moderately closely packed histiocytic cells with pale eosinophilic cytoplasm and medium-sized nuclei of variable shape, without distinct nucleoli, together with interstitial reactive lymphocytes, small numbers of eosinophils, and sparse multinucleated giant cells (Figure 3). Focal emperiopolesis was also noted. The lesions formed a circumscribed nodule without epithelioid granulomas, necrosis, or necrobiotic stromal changes.

Immunohistochemistry showed CD45 positivity in the cells within the infiltrate. CD 163 highlighted histiocytes. S100 was occasionally positive in larger histiocytes. EMA, CAM 5.2, and AE1/AE3 stains were negative. Gram and Ziehl-Neelsen stainings were negative for bacteria and acid-fast mycobacteria, respectively.

The initial biopsy report did not make a specific diagnosis and suggested a wide range of differential diagnoses, including atypical sarcoidosis, atypical interstitial granuloma annulare, unusual cutaneous Rosai-Dorfman Disease (RDD), or atypical infectious granuloma, for clinical correlation.

The patient was referred to the rheumatology, pulmonology, urology, internal medicine, and ophthalmology departments to exclude involvement in Rosai-Dorfman disease.

Ultrasound of the kidneys, ureters, and bladder, urine analysis, and renal function tests were normal apart from a mildly elevated blood urea (6.6 mmol/L, normal range 2.5-6.1). Eye examination showed age-related cataract, no macular edema, and no diabetic retinopathy. Rheumatology assessment was clinically negative with normal C-reactive protein and negative anti-nuclear antibody, anti-DNA, anti-CCP, anti-Ro, anti-La, and anti-ENP. Only the rheumatoid factor was positive at 40 IU/ml (a negative value is less than 10 IU/ml). Hypovitaminosis D (18 ng/ml) was also observed. The glycated hemoglobin (HbA1c) level was 6.5%, indicating good diabetes control. Significant laboratory findings are summarized in Table 1. Chest X-ray was unremarkable, while chest CT showed a tortuous thoracic aorta, with a dilated, possibly aneurysmal abdominal aorta.

**Table 1:** Significant laboratory findings HbA1c: glycated hemoglobin

Test	Result	Reference Range
Blood urea	6.6	2.5-6.1 mmol/L
Rheumatoid factor	40	<10 IU/ml
Vitamin D	18	30-100 ng/ml
HbA1c	6.5%	5.7-6.2% prediabetes, 6.3-7% good diabetes control

**Figure 4:**
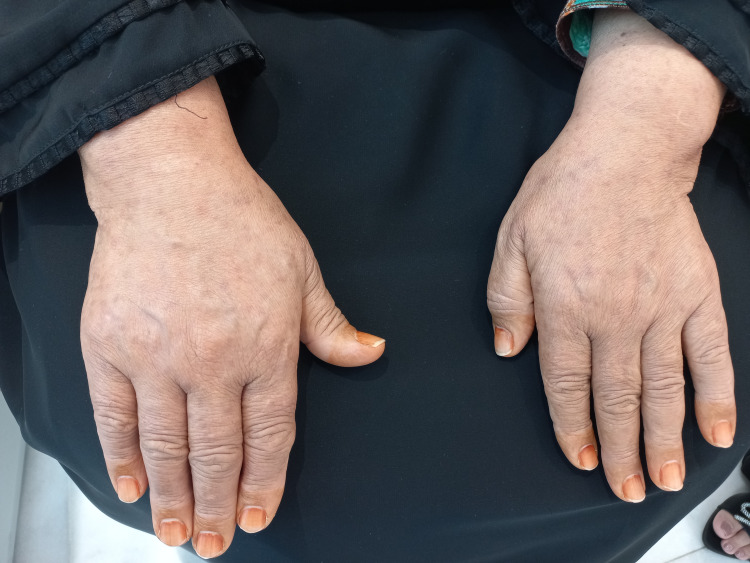
Clinical photo (after) Clinical picture at follow-up: residual macular hyperpigmentation after resolution.

On clinical follow-up visit, in December 2023, the lesions had started to involute spontaneously (Figure 4). No further clinical evaluations were requested at that time as the patient did not report any further health problems.

However, a second opinion for histopathology was obtained, and the diagnosis of GEH was established. In April 2024, the patient was contacted for another follow-up visit; however, she declined due to almost completely resolved lesions.

## Discussion

GEH is an uncommon, usually benign, type of non-Langerhans cell histiocytosis (non-LCH). Dobrosavljevic et al. (2020) reviewed 75 published cases, including their case report [3].

Focal emperiopolesis was noted, which raised the possibility of Rosai-Dorfman Disease. However, spontaneous involution contradicted this possibility. Nevertheless, Dobrosavljevic et al. (2020) described emperiopolesis in a case report of GEH [3]. Another case with myelodysplastic syndrome had concurrent Rosai-Dorfman Disease and subsequent GEH, which are usually described as two separate non-LCH disorders, while they may be closely related [4].

Other non-X histiocytoses were ruled out by clinico-histopathological correlation [5]. Multicentric reticulohistiocytosis usually manifests in patients in their thirties to fifties, with concurrent arthropathy and lesions on extremities. However, the absence of giant cells with ground-glass cytoplasm in histology excluded this diagnosis [6]. Progressive nodular histiocytoma was dismissed because of the absence of oral and conjunctival lesions and the characteristic histological profile [5,6].

Winkelmann and Müller listed the diagnostic characteristics of GEH: (a) disseminated, symmetrical, numerous lesions, usually affecting the trunk and proximal extremities and, rarely involving the mucous membranes; (b) unique flesh-coloured to blue-red discrete papules; (c) eruptive nature with spontaneous development of new crops of papules for years, without preceding trauma; (d) self-limiting with spontaneous involution of lesions, with or without residual hyperpigmented macules; and (e) histopathological features of accumulation of benign mononuclear histiocytes [7]. 

Our patient fulfilled these criteria, except for the tendency of the lesions to involve the distal extremities rather than the proximal limbs. Our patient was evaluated carefully for the possibility of underlying disease, and all the investigations were irrelevant except for her history of diabetes and her positive rheumatoid factor. Nevertheless, spontaneous resolution conflicts with the support of an underlying disease.

Jang et al. suggested that GEH can be in two forms: an undifferentiated stage that simulates other histiocytic diseases and a unique disease without a subsequent disorder [8]. However, no clinical, histopathological, or laboratory markers may predict a patient’s disease course; therefore, close monitoring is important.

The transformation of GEH into xanthoma disseminatum, multicentric reticulohistiocytosis, progressive nodular histiocytosis, and xanthogranuloma has been reported, with rarely reported cases of more than one entity co-existing in the same individual [9,10].

## Conclusions

In conclusion, we report a new case of GEH, a rare non-LCH, with some clinical and immunohistochemical characteristics. Although most GEH lesions spontaneously involute, close monitoring is mandatory to evaluate their association with underlying diseases or evolution into other types of more aggressive histiocytosis. In our case, an association with seropositive arthritis could be a possibility.
